# Development of a Novel Nested–RT–LAMP Assay for the Rapid and Accurate Coronavirus Disease-2019 Diagnosis

**DOI:** 10.1155/cjid/3343309

**Published:** 2025-04-05

**Authors:** Hadi Mirzaei, Neda Sepahi, Abdolmajid Ghasemian, Razie Ranjbar, Sahar Samsami, Yaser Mansoori, Maryam Chenari, Zahra Montaseri, Negin Namavari, Sahar Namavari, Ali Ghanbariasad

**Affiliations:** ^1^Department of Medical Genetics, School of Medicine, Zabol University of Medical Sciences, Zabol, Iran; ^2^Noncommunicable Diseases Research Center, Fasa University of Medical Sciences, Fasa, Iran; ^3^Student Research Committee, Fasa University of Medical Sciences, Fasa, Iran; ^4^Department of Virology, School of Public Health, Tehran University of Medical Sciences, Tehran, Iran; ^5^Department of Infectious Diseases, School of Medicine, Fasa University of Medical Sciences, Fasa, Iran; ^6^School of Medicine Grenada, St. George's University, St. George's, Grenada; ^7^Department of Medical Biotechnology, School of Advanced Technologies in Medicine, Fasa University of Medical Sciences, Fasa, Iran

**Keywords:** COVID-19, diagnostic tool, extraction-free approach, nested–RT–LAMP, real-time PCR, RT–LAMP, sensitivity

## Abstract

**Background and Aims:** Coronavirus disease 2019 (COVID-19), an emerging life-threatening viral disease, has rapidly spread worldwide, exerting a detrimental impact on public health. We aimed to devise an innovative platform based on the loop-mediated isothermal amplification (LAMP) method, having priorities over real-time PCR (RT–PCR) in terms of sensitivity, specificity, and low running costs.

**Methods:** To develop a novel assay, a new primer set plus four primer sets were designed targeting the N gene of the COVID-19 agent, resulting in the sensitivity reinforcement. The limit of detection (LOD) of the developed approach was determined and compared to those of the standard RT–LAMP and RT–PCR. Two hundred confirmed positive and negative samples initially tested by RT–PCR were recruited to assess the nested–RT–LAMP assay. Furthermore, for the one-step nested–RT–LAMP assay, positive samples were tested directly without the need for RNA extraction.

**Results:** The LOD of nested–RT–LAMP, LAMP, and RT–PCR were 5, 15, and 15 copies/μL, respectively. The findings of the investigation illustrated 100% sensitivity and 98% specificity for both LAMP assays. Moreover, respectively, 94% and 97% sensitivity and specificity were determined regarding the one-step nested–RT–LAMP assay.

**Conclusion:** We offered a novel approach with more sensitivity compared to RT–PCR and common RT–LAMP, not only being a simple, accurate, cost-effective alternative diagnostic tool for RT–PCR but also being able to detect asymptomatic or mildly symptomatic patients more accurately in 2 h by naked eyes.

## 1. Introduction

The outbreak of the coronavirus disease 2019 (COVID-19) pandemic has become a significant challenge for national healthcare systems, infecting millions of people, burdening daily life, and causing heavy economic losses [1]. COVID-19, with the rapid spreading rate or reproduction number (R0) being 3.28, has exerted intense pressure on hospital assistant for diagnosis beyond their capability. COVID-19' high basic R0 indicates that each infected person can spread the virus to two to three others in a susceptible population [2, 3]. This rapid transmission rate, coupled with asymptomatic carriers, facilitated widespread outbreaks globally. The rationale behind this is that asymptomatic or mildly symptomatic patients could intensify the worldwide spread of COVID-19 since they have a high chance of infecting others [4]. Early disease diagnosis, accordingly, plays an indispensable role in introducing two desired outputs: the former of which is associated with the rapid implementation of treatment for affected individuals. The latter is regarding public health measures in controlling the pandemic [5]. Real-time (RT) PCR (RT–PCR) is currently regarded as the gold standard for determining the clinical status of suspected individuals [6]. Although advantageous in terms of high specificity (up to 100%), its sensitivity is far from enough (ranging from 30% to 70% depending on viral load and on the severity and time elapsed from the onset of symptoms) [7]. An underlying repercussion of such insufficient sensitivity results is false negatives, which can occur if the viral copies are not high enough to be amplified [8]. However, it entails more than just getting false negatives; there are some other drawbacks as RT–PCR requires complex and expensive laboratory equipment, well-trained and experienced personnel, and multiple hours to complete the procedure [9]. In less affluent countries, the cost of the COVID-19 test can be almost overwhelming for governments. The overall successful population screening testing would not seem to be of practicality on account of the sheer costs involved [10]. In the current severe climate of stress, the COVID-19 outbreak cannot be ameliorated unless strong mitigation measures are taken, as a result of which, there is an unprecedented need for rapid, accurate, considerably more sensitive and convenient, cost-effective, and unsupervised diagnostic tests for COVID-19 [5].

To address this predicament, many solutions for COVID-19 detection appear to be possible, and one acid nucleic detection method which has gained popularity is called loop-mediated isothermal amplification (LAMP) [11]. LAMP diagnostic methods have been extensively used for detecting pathogenic RNA viruses as well. Detection of RNA viruses requires an additional reverse transcriptase enzyme, and this method is then referred to as reverse transcriptase LAMP (RT–LAMP) [12, 13]. Amplification and detection of the nucleic acid could be integrated and completed simultaneously in a single step, by incubating the genomic sample, four (or six) specifically designed primers, and Bst DNA polymerase in the same test tube at about 60°C to 65°C, depending on the optimal LAMP temperature [14]. Unlike PCR, LAMP could not only be carried out at a lower cost with cost-effective instruments but also brings an avalanche of interest, including a greater extent of specificity, sensitivity, and amplification efficiency [15]. By applying 4–6 primers, this assay can distinguish 6–8 regions of the target genome, leading the extent of specificity to reach at the highest level [16]. The accuracy of a diagnostic assay is chiefly in the light of sensitivity (i.e., true positive rate) and specificity (i.e., true negative rate) [17]; therefore, we decided to improve the LAMP assay and boost its sensitivity in order to identify COVID-19 infection in any stages. Instead of the two loop primers, two nested primers (innovative primers) were added to distinguish regions beyond the F3 and B3 zone of LAMP, as a result of which, the template level amplification would be bolstered and the necessary primary templates would be provided for the LAMP reaction initiation. It should detect even a minor amount of RNA template in samples. In addition, it would be less expensive, more accurate, and applicable in limited-resource settings such as developing countries. Our objective was the development of a novel nested–RT–LAMP assay for the rapid and accurate diagnosis of COVID-19.

## 2. Materials and Methods

### 2.1. Ethics Statement

Clinical samples were collected from the Vali Asr Hospital, Fasa, Iran. A total of 200 nasopharyngeal swabs (NPS) (100 negative and 100 positive selected samples stored in a virus transport medium [VTM]) were collected following the approved guidelines and relevant regulations. All subjects provided their written informed consent prior to participating in the study. The Ethics Committee of Fasa and Zabol Universities of Medical Science approved all experimental procedures (ethical code: IR.FUMS.REC.1399.132). All experiments were performed in compliance with the guidelines confirmed by this committee.

### 2.2. RNA Extraction

COVID-19 genomic RNA was extracted from the 160 μL of cases of NPS using RNJia virus kit (ROJE Technologies, Iran) in accordance with the manufacturer's protocol. After elution in RNase-free water, RNA samples were stored in aliquots of 20 μL at −80^0^C until required.

### 2.3. RT–PCR Assay

In order to detect COVID-19 in suspected cases, a commercial RT–PCR kit (nCoV RT detection kit, Sansure Biotech) was used. RT–PCR was carried out according to the manufacturers' instructions. A 50 μL of total reaction mixture contained 20 μL of RNA template, 26 μL of nCov–PCR Mix, and 4 μL of nCov–PCR–Enzyme Mix. The RT–PCR assay was performed at 50°C for 30 min for reverse transcription, followed by 95°C for 1 min and then 45 cycles of 95°C for 15 s and 60°C for 30 s using the Bio-Rad system (CFX96 Touch RT–PCR Detection Systems). RT–PCR results with CT values of more than 39 were considered negative.

### 2.4. Primer Design

A set of four specific RT–LAMP primers (F3, B3, FIP, and BIP) were designed targeting the sequence of the COVID-19 N gene reposited at NCBI (NC_045512) with the use of primer explorer V5 software (available on the Eiken Chemical Co. Ltd. website: https://primerexplorer.jp/e/). The NCBI BLAST was applied so as to design two specific primers for nested (F-nested and B-nested). To determine whether the primer sequence is specific, BLAST alignment tools, MEGA5 and Gene Runner, were implemented. The details of the oligonucleotide primers required for the amplification of the aforementioned gene are illustrated in Table 1.

### 2.5. RT–LAMP and Nested–RT–LAMP Assays

RT–LAMP reactions were carried out in a total volume of 25 μL containing 1x Isothermic Amplification Buffer, 8 mM MgSO4, 4 U of Bst DNA polymerase (Vversion 3.0 WarmStart; New England Biolabs [NEB]), 1.8 mM deoxynucleotide triphosphates (dNTPs) (ThermoFisher Scientific), 1.6 μM of BIP, 1.6 μM of FIP, 0.2 μM of F3, 0.2 μM of B3, and 0.3 μM of each F-nested and B-nested (for nested–RT–LAMP) primers. Five  microliters of test samples (negative control (NTC), extracted RNA, or samples without RNA extraction) were also added.

In order to prevent cross-contamination, the microtube cap was not opened after performing the RT–LAMP assay. On the other hand, it was necessary to add SYBR Green I to visualize or verify the amplification process. Ten-fold diluted SYBR Green I (0.5 μL, Thermo Fisher Scientific, Grand Island, New York) was added inside of the microtube cap. After closing the cap, the tube was placed at different temperatures for testing. After the test was terminated with a quick spin, the SYBR Green I dye inside the microtube cap was entered into the reaction, and if the result was positive, the color of the mixture would change to greenish-yellow. RT–LAMP and nested–RT–LAMP assays were examined at different temperatures and times ranging from 59°C to 65°C and 60 to 120 min, respectively, to establish the optimum condition. All experiments were independently replicated at least three times. After being incubated, the amplified RT–LAMP products were detected using three different methods. First, the products were observed by the naked eye under natural light and imaged via a conventional smartphone camera. All positive reactions were identified by turning their color from orange to greenish-yellow, while the indicator of the negative sample was orange. The following approach was a visual analysis of reaction tubes under UV light irradiation (UV wavelength of 302–312 nm) using a transilluminator (model UVB LTB 20  ×  20 STV, Loccus Biotecnologia, São Paulo, Brazil) coupled with a camera and connected to a computer. The dark blue color indicated negative samples and positive reactions were light fluorescents. In the third method, the RT–LAMP amplicons were analyzed by agarose gel electrophoresis (2.0%) in 1x TAE buffer employing a transilluminator. For electrophoresis analysis, 100 bp Plus DNA Ladder (Sinaclon) was used as a DNA size marker. For the nested–RT–LAMP assay, all materials were added along with F-nested and B-nested primers except for LAMP primers (BIP, FIP, F3, and B3). After some minutes at 63°C, the LAMP primers were added to the reaction and a period of time was dedicated for replication.

### 2.6. Limit of Detection (LOD)

To assess the LOD and quantification of COVID-19 genomic RNA, a serial dilution of a positive control (200 copies per μL) available in the coronavirus diagnostic kit (clontech) was prepared and examined using a standard curve. Ten positive samples were mixed (to increase the volume). Afterward, we conducted an RT test from the obtained sample to determine the copy numbers. Therefore, we considered it as the reference and LOD scores of RT–PCR, RT–LAMP, and nested–RT–LAMP, which were determined based on this reference in different concentrations.

### 2.7. Validation of RT–LAMP for COVID-19 Detection in Nasopharynx Samples

To validate the performance of the RT–LAMP and nested–RT–LAMP for the diagnosis of COVID-19 relative to RT–PCR, 200 confirmed positive and negative samples with a mean age of 52 being previously assayed by RT–PCR were obtained from the central lab of Fasa University of Medical Sciences and tested by both the developed RT–LAMP approaches. We applied a very strict policy in the selection of samples. One-hundred positive samples which were 50 females and 50 males were recruited. They also all exhibited COVID-19 clinical symptoms ranging from mild to severe. We also used 50 females and 50 males who had neither positive RT–PCR results nor any kind of COVID-19 symptoms and without any contact with COVID-19 patients to be confirmed as negative.

### 2.8. Extraction-Free Nested–RT–LAMP (One-Step Nested–RT–LAMP Assay)

For saving time and cost, we tried an approach which did not rely on a RNA extraction kit. All the confirmed positive samples, therefore, were heated at 65°C for 10 min contributing to the availability of RNA. Then, the nested–RT–LAMP method was tested on them.

### 2.9. Statistical Analysis

After evaluation of clinical samples with different molecular methods, we used the following formulas to calculate sensitivity and specificity: sensitivity = number of true positives/(number of true positives + number of false negatives) and specificity = number of true negatives/(number of false positives + number of true negatives).

## 3. Results

### 3.1. Primer Zone Mapping

**Figure 1:**
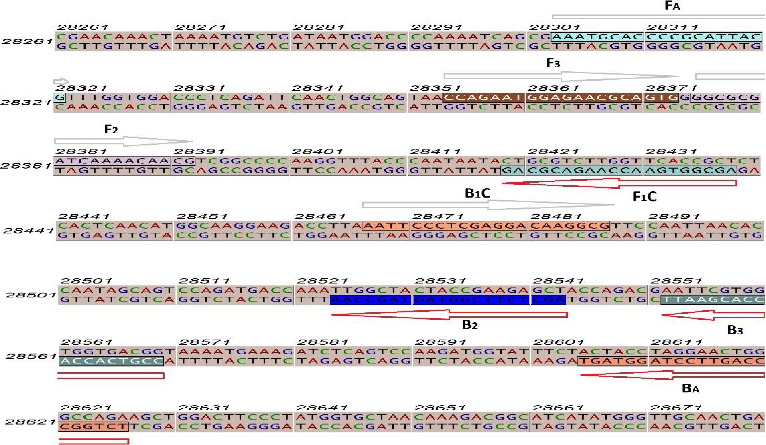
Primer design for RT–LAMP and nested–RT–LAMP assays. The *N* gene sequence of COVID-19 was used to design the primers. Arrows show LAMP primers.

The N gene for nucleocapsid protein reported to be conserved to SARS-like coronavirus was chosen [18]. The online software Primer Explorer V5 (https://primerexplorer.jp/lampv5e/index.html) was used to design the desired primers for the RT–LAMP and nested–RT–LAMP techniques represented in Figure 1. Two nested primers had the same temperature melting (TM) with F3 and B3. Moreover, being checked by the BLAST of NCBI, MEGA5, and GENE RUNNER, primer specificity was assured. Notably, sequences selected for the current study did not match the other members of the coronavirus family and influenza viruses' sequences.

### 3.2. The RT–LAMP and Nested–RT–LAMP Optimization

For the RT–LAMP assay, first, we tested different temperatures ranging from 59°C to 65°C and 63°C to be valid for selection. In addition, a time duration between 60 and 120 min was evaluated to select the optimized one, where 120 min was the optimum time to reach the highest sensitivity.

**Figure 2:**
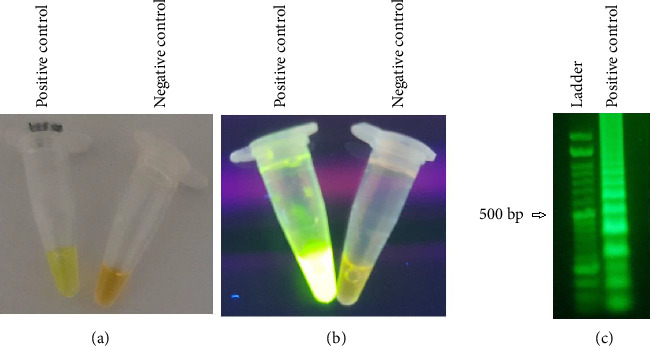
The color change of nested–RT–LAMP products in order to distinguish infected cases. The color transforms in the presence of COVID-19 agents from orange to greenish-yellow.

Regarding the novel nested–RT–LAMP approach, first, two F-nested and B-nested primers and also all necessary materials for the reaction such as RNA templates were added. Again, we attempted different time periods for adding the remaining primers, which were 5, 10, 15, and 20 min, and among them, 10 min was the proper time. After 10 min at 63°C, the LAMP primers were added to the reaction and a period of time was dedicated for replication. The target RNA was added to the reaction, and amplifications were performed in different amounts of time; the maximum sensitivity was obtained as long as 2 hours. After the test was finished with a quick spin, the SYBR Green I dye inside the microtube cap was entered into the reaction, and the color remained unalterable without amplification, while amplified products caused the color shift into a greenish-yellow, indicating positive samples (Figure 2).

### 3.3. The LOD Determination

**Figure 3:**
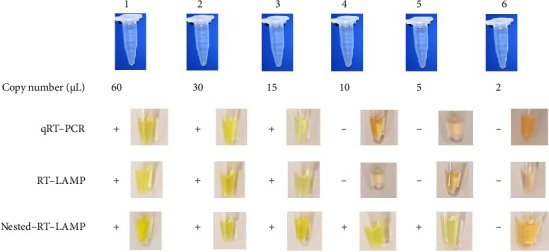
The comparison of three studied approaches (nested–RT–PCR, RT–LAMP, and real-time PCR) in terms of LOD. All were assayed in the presence of 60, 30, 15, 10, 5, and 2 viral copy numbers/μL. RT–LAMP and real-time PCR could detect only with 15 viral copy numbers/μL, while, the LOD of nested–RT–PCR decreased to 5. However, at the two copy numbers of the virus, none of the methods were positive and lacked this sensitivity level.

The LOD of the nested–RT–LAMP was calculated and compared with the other molecular techniques performed in this research (RT–PCR and RT–LAMP). A serial dilution of a positive control available in the coronavirus diagnostic kit was prepared and examined to get to this end. The same range of LOD was obtained for conventional RT–PCR and RT–LAMP (15 copies/μL); on the other hand, our innovative method showed a three-fold detection limit of 5 copies/μL, indicating the considerable enhancement of assay's sensitivity (Figure 3).

### 3.4. Tests Performance on Clinical Samples

Our novel nested–RT–LAMP assay was validated by comparing its sensitivity and specificity with a validated RT–PCR method as the gold standard technique. In this comparative study, therefore, we used a total of 200 clinical samples (100 confirmed positive and 100 negative specimens) in parallel, taken by NPS from patients referred to Vali Asr Hospital in a standard way. These samples had been initially diagnosed by conventional RT–PCR with different cycling thresholds (Table 2), and then common RT–LAMP and nested–RT–LAMP were implemented to test the same samples. The findings indicated that all affirmed positive samples were also found to be positive by RT–LAMP and nested–RT–LAMP. Hence, the sensitivity of both approaches reached 100%, showing no false negatives, whereas those experienced the color of 2% of negative controls into a greenish-yellow, decreased the specificity to 98%. Furthermore, we used another approach to save time, energy, and costs in which RNA extraction was omitted. The sensitivity and specificity of one-step nested–RT–LAMP (with no requirement for RNA extraction) were 94% and 97%, respectively (six false negatives and three false positives) (Tables 2 and 3). Six false negative samples belonged to the two last classes at Table 2 (CT > 31). The CT value of five of them was between 36 and 39 and one was between 31 and 35.

## 4. Discussion

The world comes across an ever-increasing concern due to the COVID-19 pandemic that emerged since 2019, which exerts a burden on society and public health systems as it has been spreading around the globe rapidly [19, 20]. As rapid detection of COVID-19 agents is pivotal, molecular detection tools such as RT–PCR cannot be utilized in resource-free settings, as sophisticated equipment and well-trained personnel are needed for mass screening. Therefore, we took the lead to develop a LAMP assay called nested–RT–LAMP, which is not only able to diagnose patients even in the initial days of infection due to its high sensitivity but is also specific, efficient, rapid, cost-effective, and applicable in any setting whether situated in wealthy regions or destitute parts and remote areas throughout the world [21, 22].

The characteristics of COVID-19 during infection, including increase, infectivity, and decline, greatly impact viral detection tests of different sensitivities [23]. The RT–LAMP method has been used to detect coronavirus family members, including COVID-19 and MERS agents (10, 11). While false negative results of RT–PCR exacerbate the current global condition, the majority of diagnostic tools are in the verge of a transformation in order to get improved in terms of accuracy and sensitivity. In the RT–LAMP technique, primers are the major driver of controlling its sensitivity and performance because other parts of the test are optimized and stable. Therefore, obtaining the optimal RT–LAMP primer sets to detect and identify individuals affected by this deadly virus is of crucial importance for adopting the preferred or accurate approach to use for large field screening of COVID-19 patients [24]. Primers selected for a target genomic region and considering the notion that the temperature of these primers should be set with the optimum working temperature of the DNA polymerase is necessary. As a result, designing primer sets is the most challenging step while developing RT–LAMP assays [25]. Apart from designing optimum primers, we made unlimited efforts to add two more primers in order to devise our novel LAMP assay as their melting temperature must be matched with the other primers and contribute to overcoming previous drawbacks. The primers that passed the BLAST search were indicated to be highly specific, providing a low chance for the amplification of unspecific products (false positives). After screening out the best primers, the next important stage in the assay optimization was adding nested primers first. With a ten-minute delay, the remaining primers were included, which helped give the amplification reaction sufficient time to replicate more genomic target COVID-19 regions. This resulted in reinforced sensitivity and more efficient amplification compared to common LAMP assay and RT–PCR. Another criterion for determining the optimum sensitivity and performance of RT–LAMP is selecting an appropriate genomic target zone. We decided to design our primers based on the N gene of COVID-19 being rooted in the fact that the coronavirus' N protein does not contain the glycosylation site and carries distinctly unaltered immunological entities [26]. In addition, being the most abundant protein during the infection could be regarded as a definite plus, as well as this protein is encoded by a highly conserved gene [18]. Another study by Yang et al. confirmed this selection, in which through these three candidate genes (the ORF1ab gene, E gene, and N gene) of COVID-19, N gene–based RT–LAMP assay was reported as the most sensitive locus with the fastest amplification speed. It was reported that its sensitivity was over 80 times as high as the overall sensitivity [27]. Our developed LAMP assay, nested–RT–LAMP, showed a stark contrast to common LAMP and conventional RT–PCR considering LOD level. Nested–RT–LAMP could identify infection in the presence of RNA template molecules with as few as 5 copy numbers, whereas the LOD of the other used methods was 15 copies/μL. The sensitivity achieved by both LAMP tests was in 100% agreement with the RT–PCR results outlining no false negative while evaluating clinical samples. Owing to the high sensitivity, LAMP assays caused false positive results due to carry-over or cross-contamination throughout the entire course of examination [28], representing two false positives that decreased specificity to 98%. Lu et al. developed a LAMP assay using a mismatch-tolerant amplification technique in which the lowest concentration detected was 118.6 copies of COVID-19 RNA per 25 μL reaction (4.7 copies/μL). In addition, the sensitivity was similar to that of our LAMP assay with high specificity [29]. Another one-step colorimetric COVID-19-LAMP developed by Chow et al. indicated 96.88% and 98.96% sensitivity for NPS at 60 and 90 min, respectively, in relation to RT–PCR assay as the gold standard. The specificity, moreover, was perfect without any false positive results. This simple approach could be interpreted by a color change and could detect 42 copies/reaction of COVID-19 RNA [30]. Another study working on a LAMP assay showed 91.4% and 99.5% sensitivity and specificity, respectively [31]. One research that detected COVID-19 by RT–fluorescence LAMP (RT–fLAMP) inferred 97% and 100% sensitivity and specificity. However, the sensitivity figures experienced a dramatic fall without RNA extraction using NPS and saliva, i.e., 71% and 47%. It could be concluded that the RNA extraction process might be vital in this detection system [32]. We also tested all confirmed RT–PCR samples directly without exploiting RNA. The NPS was heated at 65^0^C because this action could help degrade the viral membrane. It is true that the obtained sensitivity was nothing like as low as the unextracted RNA RT–LAMP. Nevertheless, there was a slight decrease in sensitivity and specificity percentages calculated at 94% and 97%, respectively. However, its sensitivity was higher in comparison to Lali's developed extraction-free RT–LAMP assay, which was 86%. Their nonsophisticated method was able to detect 2.95 copies/μL with 100% specificity [33]. As a matter of fact, in the current research, six false negative samples' CT values were above 31. One of them was between 31 and 35 and the rest were above 36. This means that the extraction-free RT–LAMP assay can be applicable for the samples with CT values of 30 and lower.

The ongoing COVID-19 pandemic necessitates rapid and accurate diagnostic tools. RT–PCR, the current gold standard, has limitations in sensitivity and accessibility. We present a novel nested–RT–LAMP assay targeting the N gene of SARS-CoV-2, offering superior sensitivity compared to RT–PCR and standard RT–LAMP. This assay achieved a LOD of 5 copies/μL, potentially detecting infections earlier and reducing false negatives. In addition, the possibility of a one-step extraction-free approach further enhances its feasibility in resource-limited settings. While the extraction-free method demonstrated slightly lower sensitivity and specificity (94% and 97%, respectively) compared to the standard extraction-based method (100% and 98%), it still provides a valuable option for the rapid and convenient testing. The reduced performance might be attributed to factors such as incomplete RNA release during the heating process or the presence of inhibitory substances in the clinical samples [34]. Further optimization of the extraction-free protocol, including adjusting the heating temperature or adding additional lysis buffers, could potentially improve its sensitivity and specificity. This novel assay holds promise for improved COVID-19 diagnosis and control efforts, particularly in settings where rapid testing is essential and resource constraints limit the availability of traditional extraction methods.

A considerable number of advantageous qualities of nested–RT–LAMP could be associated with its convenience using a single bathwater or a heat block rather than thermocyclers or PCR machines requiring sophisticated core laboratories and well-trained technicians [9]. It should be also pointed out that the developed assay could be judged only by naked eyes in 2 h just by color change. All these features make this approach considerably less expensive as it does not need any specific facilities plus professionals [9, 29, 30]. As a portable and simple test, nested–RT–LAMP could be implemented in deserted or remote areas in which providing diagnostic amenities regarding COVID-19–infected subjects is impossible particularly in developing countries.

In the light of the abovementioned analysis, our developed LAMP assay is considered as one of the approaches with ultimate sensitivity and also might be enhanced by targeting more conserved genomic viral regions of COVID-19. However, further research is required to bolster the specificity in order to reach 100%. While the demerit of the marginal fall in assay's specificity in the path of COVID-19 outbreak control would be overshadowed by the merits of significant high sensitivity and would perhaps not detract from this achievement. One limitation of nested RT–LAMP assay includes the high contamination rate or cross-contamination which can be overcome by implementations described in this study.

## 5. Conclusion

Our developed simple LAMP assay, nested–RT–LAMP, seems to ameliorate the exorbitant costs that the available detection tests such as RT–PCR are involved in, and the expenses would become more affordable in a condition in which the world is riddled with COVID-19. It could facilitate the process of testing without any requirement for special molecular laboratories with sophisticated facilities, maintenance, or skilled biologists, which could be interpreted just by an unaided eye. More importantly, the LOD of nested–RT–LAMP was over three times as great as typical LAMP and RT–PCR, which would identify the patients with too low viral loads more rapidly and conveniently. The rationale behind such high sensitivity leading to 100% was designing two more primers and obtaining 98% specificity. Therefore, this assay could detect COVID-19 patients in all disease stages (early, progressive, and recovery). The sensitivity and specificity of this approach without RNA extraction were fractionally less than the nested–RT–LAMP with the extracted RNA, which were 94% and 97%, respectively.

## Figures and Tables

**Table 1:** Primer sets designed for both LAMP and nested–RT–LAMP assays.

F-nested	AAATGCACCCCGCATTACG
B-nested	TCTGGCCCAGTTCCTAGGTAGT
F3	CCAGAATGGAGAACGCAGTG
B3	CCGTCACCACCACGAATT
FIP	AGCGGTGAACCAAGACGCAGGGCGCGATCAAAACAACG
BIP	AATTCCCTCGAGGACAAGGCGAGCTCTTCGGTAGTAGCCAA

**Table 2:** Number of patients and CT value of real-time PCR.

Number of patients	Cycle of threshold
20	10–15
20	16–20
20	21–25
15	26–30
12	31–35
13	36–39

**Table 3:** LOD, sensitivity, and specificity of the studied approaches.

Techniqes	LOD	Sensitivity	Specificity
Real-time PCR (reference)	15 copies/μL	100%	100%
RT–LAMP	15 copies/μL	100%	98% (two false positives out of 100)
Nested–RT–LAMP	5 copies/μL	100%	98% (two false positives out of 100)
Extraction-free nested–RT–LAMP	—	94% (six false negative out of 100)	97% (three false positives out of 100)

## Data Availability

No underlying data were collected or produced in this study. All data have been included in the manuscript file.
